# The role of the positive emotional attractor in vision and shared vision: toward effective leadership, relationships, and engagement

**DOI:** 10.3389/fpsyg.2015.00670

**Published:** 2015-05-21

**Authors:** Richard E. Boyatzis, Kylie Rochford, Scott N. Taylor

**Affiliations:** ^1^Case Western Reserve UniversityCleveland, OH, USA; ^2^Babson College, Babson Park, MAUSA

**Keywords:** positive emotional attractor, vision, shared vision, leadership, engagement, organizational citizenship

## Abstract

Personal and shared vision have a long history in management and organizational practices yet only recently have we begun to build a systematic body of empirical knowledge about the role of personal and shared vision in organizations. As the introductory paper for this special topic in *Frontiers in Psychology*, we present a theoretical argument as to the existence and critical role of two states in which a person, dyad, team, or organization may find themselves when engaging in the creation of a personal or shared vision: the positive emotional attractor (PEA) and the negative emotional attractor (NEA). These two primary states are strange attractors, each characterized by three dimensions: (1) positive versus negative emotional arousal; (2) endocrine arousal of the parasympathetic nervous system versus sympathetic nervous system; and (3) neurological activation of the default mode network versus the task positive network. We argue that arousing the PEA is critical when creating or affirming a personal vision (i.e., sense of one’s purpose and ideal self). We begin our paper by reviewing the underpinnings of our PEA–NEA theory, briefly review each of the papers in this special issue, and conclude by discussing the practical implications of the theory.

## Introduction

For many years practitioners and academics alike have argued that the creation of a vision, be it at the individual, team, or organizational level, motivates people to action and inspires them to reach beyond their current state. Oddly, empirical evidence pertaining to the antecedents and consequences of vision remains fragmented and scarce. There is not an agreed upon definition of the concept of vision (Kantabutra and Avery, 2002), nor do we understand the underlying mechanisms that influence how a person, team, or organization arrives at an effective vision. This special edition of *Frontiers in Psychology* addresses the importance and impact of personal and shared vision.

As an introduction to the papers in this special issue, we present a series of theoretical propositions regarding the existence and critical role of two psycho-physiological states which we believe are intricately involved in the creation and realization of a personal vision or shared vision: the Positive Emotional Attractor (PEA) and the Negative Emotional Attractor (NEA). Using complexity theory, we argue that these two states are strange attractors, each characterized by three dimensions: (1) positive versus negative emotional arousal, (2) hormonal arousal; and (3) neurological activation (Boyatzis, 2008). To our knowledge, our PEA–NEA theory is one of the first theories that brings together and integrates early work on emotion and the self with recent advances in physiological measurement and neurological activity. Additionally, this is one of the first papers that addresses the underlying mechanism of the visioning process and sheds light on how elements of the process of arriving at a vision consequently impact the content of the vision that is developed – which we know from existing research, impacts the effectiveness of that vision (Kantabutra and Avery, 2010).

In this paper we make three key arguments: (1) a personal vision based on an ideal self is required if the vision is to lead to sustained and desired change; (2) in order to create a personal vision based on an ideal self, or among others, a shared vision, a person must be in the PEA; and (3) while the NEA is required to move a person from vision to action, a person must spend significantly more time in the PEA in order to achieve sustained desired change. We begin by examining the theory of regulatory focus to build an argument as to why the content of a vision and process of visioning are critical components of arriving at an effective vision. We then integrate literature from the fields of emotion, psychology, physiology, and neuroscience to introduce two theoretical constructs: the PEA and NEA. Following this, we link the PEA and NEA to personal and shared vision and explain why the PEA is necessary in order to formulate an engaging vision that will motivate sustained and desired change. Finally, we address the role of the NEA and the necessary balance between the PEA and NEA that is required to move a person closer to their vision. After presenting our propositions, we provide a brief introduction to the papers included in this special issue. We conclude with a discussion of the practical implications of PEA–NEA theory and directions for future research.

## Vision and Positive and Negative Attractors

### Contents and Process of Vision

Whether at the individual, team, or organizational level, visions, and shared visions are generally developed to create motivation to move from a current state to a desired end state. Regulatory focus theory proposes two different ways in which a person may approach an ideal state: a “promotion focus” and a “prevention focus” (Higgins, 1997). Higgins (1997, p. 1282) argues that when faced with a discrepancy between a current state and an ideal state, an individual with a promotion focus will be motivated to approach the desired end state based on concerns with “advancement, growth, and accomplishment.” Conversely, a person with a prevention focus will be motivated to approach the desired end state based on concerns with “protection, safety, and responsibility” and avoid risks and danger. Individuals with a promotion focus experience pleasure and pain as a result of the presence or absence of positive outcomes while individuals with a prevention focus experience pleasure and pain as a result of the presence or absence of negative outcomes.

Higgins proposed three variables that are responsible for the regulatory state a person experiences. A promotion focus is aroused by a focus on nurturance needs, strong ideals, and “gain/no-gain” situations. Conversely, focusing on security needs, strong “oughts” and “non-loss/loss” situations arouse a prevention focus. Based on this, visions that are founded on nurturance needs, strong ideals, and “gain/no-gain” situations will elicit a promotion focus while visions founded on security needs, strong “ought’s,” and “non-loss/loss” situations will elicit a prevention focus. Thus, the basis of a vision becomes a critical variable in influencing the regulatory state that will drive the individual toward their vision. In the following section we distinguish between the *ideal self* and the *ought self* and argue that for a vision to lead to sustained and desired change it must elicit a promotion focus, and thus be based on an ideal self rather than an ought self.

The development of alternate future scenarios, also called “prospection” (Gilbert and Wilson, 2007), is a cognitive process with profound emotional features that enables us to transcend behaviorism and cognitive determinism (Seligman et al., 2013). Gilbert and Wilson (2007, p. 1351) defined prospection as, “…our ability to ‘pre-experience’ the future by simulating it in our minds.” Current research on prospection includes neurological and simulation studies as well as forecasting, and highlights the distinction between ‘goal directedness’ and ‘purpose and dreaming’ (Gilbert and Wilson, 2007; Seligman et al., 2013). The former is aiming for a target and the latter is aspirational and significantly less specific. In this paper, we focus on the purpose and dreaming aspect of prospection as the critical ingredient in developing a personal or shared vision.

At the center of the concept of vision is that the desired images of the future, or a hoped for future, helps create, or remind people about their sense of purpose. Deeper than goals or strategy, vision can provide a sense of mission. This sense of purpose has been shown to help with mortality (Hill and Turiano, 2014) and increased career commitment over time (Dobrow Riza and Heller, 2015). One of the papers in this Special Issue (Buse and Bilimoria, 2014) shows that sense of purpose as part of a female engineer’s personal vision, or ideal self, significantly predicts career engagement and career commitment in STEM fields (i.e., science, technology, engineering, and math careers).

Leadership can help others find direction and purpose through vision. A leader emphasizing vision elicits more adaptability and openness in those within the organization (Griffin et al., 2010). For example, aspiring to help others and promote health can be an inspiring vision for hospitals. Carton et al. (2014) showed that invoking a desired image in the future and selected values inherent in that image was the most motivating and predictive of organizational performance. This stands in contrast to a statement by some hospitals that their desire is to provide the best health care, which is more of a goal than a vision, or they skip that entirely and focus on budgets and showing financial sustainability. The latter communicates to patients and their families, doctors, nurses, and staff and potential donors that their real intent is to make money. While fiscal responsibility and financial survival is a necessity, it is limiting and does not appear to generate the kind of excitement derived from inspiring vision statements (Carton et al., 2014).

### The Contents of Visions: Ideal Self Versus Ought Self

Within the broader psychological literature, the ideal self could be considered as a subset of possible selves (Markus and Nurius, 1986, 1987; Martinez, unpublished dissertation proposal), which are described as self-schemas derived from representations of future selves that capture the cognitive components of a person’s “hopes, fears, goals, and threats” (Markus and Nurius, 1986, p. 955). However, in contrast to the possible self, the ideal self discussed in this paper and in a number of papers in this *Special Issue* is not concerned with negative possible selves, but rather a version of a future self that is consistent with our core values, aspirational and also inspirational. Additionally, the focus is on the ‘ideal’ rather than the ‘probable.’ In this regard, the ideal self as we see it is perhaps most consistent with the early work of Levinson (1978) who conceptualized ‘the Dream’ as an imagined self that represents a variety of conscious and unconscious desired states, aspirations, and values. One major theoretical distinction between PEA/NEA and promotion/prevention focus is that we do not consider “goals” a part of the PEA state. This rationale will be explained later. In that sense, Higgins’ promotion focus is more concentrated on goals and, in his words, “ambitions” rather than the dream and aspirations of the PEA. It is also important to note the ideal self in this paper is distinct and in contrast to Rogers (1951) ideal self that is defined as “how I *should* be” [emphasis added]. How a person believes that they should be is closer to our conceptualization of the ought self discussed below.

### Distinguishing between Ideal and Ought Self

Boyatzis and Akrivou (2006) define the ideal self as a psychological component of the self that is partially conscious and partially unconscious and is both privately conceptualized and socially influenced. The ideal self is comprised of three main components: (1) an image of a desired future that is (2) emotionally fuelled by hope, and (3) reflects a person’s core identity.

The manifestation of the ideal self is a personal vision that articulates a person’s “dreams, aspirations, and fantasies” (Boyatzis and Akrivou, 2006, p. 626). In contrast to the general possible self that is, by definition, purely cognitive, we consider emotion as a part of each of the components of the ideal self. We believe that the deep and fundamental alignment of the ideal self with a person’s core identity, values, goals, and aspirations enables the arousal of hope and efficacy, without which positive emotion would not be manifested and, as will be discussed later, a person would not be in the PEA.

In contrast to the ideal self, the ought self is someone else’s desire or interpretation of what a person’s ideal self should be (Boyatzis and Akrivou, 2006). While it is possible that a person’s ought self and ideal self are not in conflict, our experience suggests that this is a rare occurrence. Boyatzis and Akrivou (2006, p. 628) warn that working toward an ought self will lead to feelings of betrayal, frustration, and anger as a result of realizing that the person had wasted time and energy “in pursuit of dreams and expectations that they were never passionate about”. One caveat to this point is in the case that the ought self is fully internalized and integrated into the ideal self. In this case a person is able to have fully accepted an ought self “by bringing them into harmony or coherence with other aspects of their values and identity” (Deci and Ryan, 2000, p 236). In this case, it is likely that an external influence is internalized so deeply that over time it actually changes a person’s core identity (e.g., certain religious movements). As a person’s core identity changes, the ought self aligns with the ideal, reconciling any conflict between the two. While we believe that this situation is a rare occurrence, it highlights the intricate relationship between ideal and ought selves and the difficulty that many people experience when trying to separate the two.

In line with regulatory focus theory, we believe that developing a personal vision based on an ideal self results in a promotion focus, thus individuals are motivated to approach situations that are congruent with their personal vision and avoid those that are not (Higgins, 1997). The ideal self is concerned with growth, ideals, hope, congruence in harmony with one’s values – the three variables that Higgins’ attributes to a promotion focus, with the exception of goals and ambition. In contrast, personal visions that are based on an individual’s ought self are based on security needs and non-loss situations; such visions are consistent with a prevention focus. There is some empirical evidence that supports our claim. Specifically, Higgins et al. (1994) found that a person’s concern with approach is greater for the ideal than the ought self-regulation, while a concern with avoidance was greater for ought than the ideal self-regulation.

Although the versions of the self used by Higgins et al. (1994) are not entirely consistent with the ideal self proposed in this paper, the underlying principle remains the same. Further support for our claim can be found in a recent dissertation study (Passarelli, unpublished doctoral dissertation) that found participants that were coached around the PEA (which we later argue is an essential antecedent of an ideal self vision) demonstrated an ‘attentive-interested’ emotional state that is consistent with an approach motivation. In contrast, those coaching around the NEA (which we later argue is a likely consequence of an ought self vision) demonstrated an ‘attentive-alert’ state that was indicative of the vigilant avoidance state of a prevention orientation.

The relevance of the prevention and promotion focus to this paper is that we believe that in order for a vision to be effective – that is lead to sustained and desired change – it must be based on an ideal self rather than an ought self. We believe that this requires a promotion focus for two key reasons. First, while a prevention focus might spur a person to action to achieve short-term outcomes, any behavioral change approached from a loss/non-loss situation is unlikely to be maintained in the long term. Ironically, change actually requires a willingness to ‘lose’ a current state in order to move to a new, desired state. This point reflects the famous quote from Jim Collins: “Good is the enemy of great” (Collins, 2001, p. 1). In other words, if we approach change with a prevention focus, at best we will maintain the ‘good’ but we will not move beyond it. As discussed above, a vision based on an ought self elicits a prevention focus based on a loss/non-loss framing. This type of vision will not allow a person or organization to move to a new desired state. Rather, in order for a vision to be effective, the vision must be based on a gain/no-gain framing characteristic of a promotion focus and the ideal self.

Second, a key enabler of the motivation gained from the ideal self is efficacy and hope (Boyatzis and Akrivou, 2006). Efficacy is derived from the fundamental alignment of the person’s core identity with their ideal self and manifest vision. This could also be termed ‘internalization’ of the vision in a cognitive and affective manner. This core and fundamental alignment does not occur when a vision is based on an ought self, as the ought self is reflective of someone else’s perception of your identity and values rather than your actual identity and values. Without the fundamental motivational drivers of efficacy and hope, a vision is unlikely to lead to sustained and desired change. In sum, we propose the following.

Proposition 1: Visions must be based on an ideal self rather than an ought self in order to produce sustained and desired change.

### Overview of the Positive and Negative Emotional Attractors

The PEA and NEA are two distinct psycho-physiological states comprised of distinct emotional, psychological, physiological, and neurological characteristics that create “a force around one’s thinking, feeling, and behaviors” (Passarelli, unpublished doctoral dissertation, p. 20). A summary of the characteristics associated with each state is provided in **Table 1** below. The relationship between these neural networks with the other components of the PEA–NEA are not likely to be linear or a simple correspondence. However, there is a growing body of evidence that shows that PEA experiences activate a distinct neural network called the default mode network (DMN), while NEA experiences suppress the DMN (Jack et al., 2012; see also Passarelli, 2015; this issue). The DMN is a neural network that primarily includes simultaneous activation of the prefrontal cortex (MPFC), the medial parietal cortex (MPC), posterior cingulate cortex (PCC), and the right temparo-parietal junction (rTPJ; Jack et al., 2012). We will discuss the DMN in more detail later.

**Table 1:** Characteristics of positive and negative emotional attractors (PEAs and NEAs) (adapted from Boyatzis, 2013 and Passarelli, unpublished doctoral dissertation).

	Positive emotional attractor (PEA)	Negative emotional attractor (NEA)
Physiological	Greater parasympathetic influence	Greater sympathetic influence
	Release of oxytocin and vasopressin associated with social bonding	Release of epinephrine and norepinephrine to mobilize defenses; release of cortisol
	Decreased blood pressure	Increases pulse, blood pressure, and rate of breathing
	Higher heart rate variability	Lower heart rate variability


Neurological	Default mode network (DMN) neurogenesis	Task positive network (TPN) Inhibited neurogenesis
Emotional	Positive affect: hope, joy, amusement, elation	Negative affect: defensiveness, guilt, shame, fear, anxiety
Cognitive	Enhanced working memory and perceptual openness	Decreased executive functioning; Limited field vision/perception
	Global attention	Local attention
	Promotion focus	Prevention focus
Relationships	Learning orientation Resonant (in tune with each other)	Performance orientation Dissonant (out of sync or distant)

The physiological distinctions listed in **Table 1** have yet to be validated, although initial studies strongly suggest that physiological activation is an important part of the PEA (Passarelli, unpublished doctoral dissertation) specifically, and more generally, positive affect (see Table 1 in Heaphy and Dutton, 2008 for a review). The neurological distinctions shown in **Table 1** have been validated in two fMRI studies (Jack et al., 2013; Passarelli et al., 2014), as have the emotional distinctions (see Howard, 2015; Passarelli, 2015). The cognitive distinctions listed in **Table 1**, with the exception of the memory and field of vision were validated by Passarelli et al. (2014). The relationship distinctions listed in **Table 1** were validated in Boyatzis et al. (2012) in a study of neural activations from follower-leader relationships. We acknowledge that these initial validation efforts are just the beginning of an ongoing validation and replication process, however, **Table 1** offers a set of underlying theoretical distinctions that can continue to be tested.

Boyatzis (2008) argued that the PEA and NEA are strange attractors (Lorenz, 1963; Erdi, 2008). As strange attractors, the PEA and NEA allow for multiple trajectories of behavior and emotions within each state, respectively, however, once in either the PEA or NEA, a person will generally return to a similar, although not identical, state as they started (Manson, 2001). This idea is similar to Fredrickson’s broaden and build theory of emotions, which posits that positive emotions are self-reinforcing due to the psychological and physiological resources that are created when positive emotions are experienced.

In other words, PEA and NEA are self-regulating states; therefore, once a person is in either a PEA state or a NEA state, the person will remain in that state until a tipping point provokes a shift to the alternate state (Boyatzis, 2008). Self-regulating systems are inherently homeostatic, therefore unless the system is perfectly efficient (which humans are not; Ferber, 1999), deterioration will occur over time. We know that negative emotions are stronger than positive emotions (Baumeister et al., 2001); as a result, it seems fair to assume that unless the PEA state is actively maintained over time, we will eventually move toward the NEA even without a salient tipping point.

Tipping points may be reached due to an emotionally salient event or a high dosage of less salient events. For example, a person who is in the NEA may move to the PEA as a result of a particularly joyful event such as the birth of a child. Alternatively, a person may experience a number of positive events over a longer period of time that gradually reduces the intensity of the NEA, which consequently allows the person to move to the PEA. This point becomes relevant later in our paper when we argue that in order to create a vision that will invoke sustained and desirable change, a person must be in the PEA, and the process of creating this vision creates a dosage effect that can move a person from the NEA to the PEA.

### Positive Emotional Attractor

First and foremost, the PEA is characterized by varying degrees of positive emotions. Emotions may be defined as “multicomponent response tendencies that unfold over relatively short time spans… [resulting in a] cascade of response tendencies manifest across loosely coupled component systems, such as subjective experience, facial expression, cognitive processing, and physiological changes” (Fredrickson, 2001, p. 218; for a discussion of the nuances of emotion and affect, see Fredrickson, 2001). Positive emotion, therefore, refers to discrete emotions that we use to describe or express our response to a pleasant experience or object. Examples of positive emotions include joy, interest, amusement, and love (Fredrickson, 2001).

The benefits of positive emotions have been a focus in behavioral and social science research over the past decade, particularly since the explosion of the positive psychology and positive organizational scholarship movements. Attributes of positive emotion that appear to be particularly relevant to the process of articulating an effective vision include higher levels of optimism about the future (Bower and Forgas, 2001), greater perceptual openness (Fredrickson and Branigan, 2005; Talarico et al., 2009), and openness to behavior change (Janig and Habler, 1999). Additionally, positive emotional states increase the likelihood of altruistic, helpful, cooperative, and conciliatory behavior (Insel, 1997; Barsade and Gibson, 2007) and improved decision making (Chuang and Lin, 2007).

While positive emotion is a necessary component of the PEA, positive emotion alone will not induce a PEA state. A person’s positive emotion must also be accompanied by the arousal of the parasympathetic nervous system (PNS) and activation of the DMN. The PNS is a subset of the autonomic nervous system that supports our ‘rest and digest’ functions, immune system, cardiovascular health, and the neuroendocrine system (Uchino et al., 1996). The PNS also supports social engagement. Arousal of the PNS arouses the vagus nerve, and consequently, triggers the release of a number of hormones including oxytocin in women and vasopression in men (Insel, 1997; Schulkin, 1999; Kemp and Guastella, 2011). It is the release of these hormones that is largely responsible for the health benefits commonly associated with positive emotions including general wellbeing (Heaphy and Dutton, 2008), improved immune system functioning (Mahony et al., 2002), faster physical recovery following surgery (Carver and Scheier, 1993), lower risk of angina and heart attacks (Kubzansky et al., 2001), and lower risk of depression (Davis et al., 1998).

Finally, in conjunction with positive emotion and arousal of the PNSs, emerging evidence from the cognitive neuroscience domain suggests that the PEA is also associated with the DMN. Specifically, two fMRI studies that examined the neurological activation during coaching interactions showed significant activation of areas of the DMN when participants were coached around the PEA rather than the NEA (Jack et al., 2013). A separate study that asked participants to recall memories of resonant (PEA) leaders revealed consistent findings – recalling memories of resonant leaders activated the parts of the DMN, while recalling memories of dissonant leaders activated the task positive network (TPN; Boyatzis et al., 2012).

The DMN has been associated with similar benefits as positive emotions and, more specifically, the PEA, including higher creativity and openness to new ideas (Raichle et al., 2001; Andrews-Hanna et al., 2010; Mars et al., 2012); emotional self-awareness (Ochsner et al., 2005; Schilbach et al., 2008), and social cognition (Schilbach et al., 2008; Jack et al., 2012; Mars et al., 2012).

Activation of the DMN may be directly linked to arousal of the PNS through the ventral medial prefrontal cortex (VMPFC, Eisenberger and Cole, 2012). The relationship, as mentioned earlier, is not linear and the time to activate or arouse neural systems versus hormonal systems varies. In addition, causality is likely both directions. In a follow-up, replication study of Jack et al. (2012), the VMPFC was significantly activated in a random effects analysis by two or three PEA sessions in contrast to one or no PEA coaching session (Jack, personal communication, March 3rd 2014).

In sum, the PEA is a psycho-physiological strange attractor that is derived from unique combinations of positive affect, PNS arousal, and activation of parts of the default mode network (DMN). The positive benefits of the PEA are realized as a result of the relatively stable nature of the strange attractor that explains the self-reinforcing nature of the PEA. Once the PEA has been activated, it acts as a positive force and guide on our subsequent thoughts and behavior (Boyatzis et al., 2013, p. 162).

### Negative Emotional Attractor

In stark contrast to the PEA, the NEA is characterized first and foremost by negative emotions such as fear, anxiety, sadness, anger, disgust, and despair (Levenson, 1992; Fredrickson, 2001). It is generally accepted across a broad range of literature that negative emotions are stronger than positive emotions – that is, negative events produce “larger, more consistent, more multifaceted, or more lasting effects than positive events” (Baumeister et al., 2001, p. 325). Baumeister et al. (2001) argue that this is a necessary function of human beings as negative emotions allow humans to be highly adaptable and thus, facilitate human survival. As Boyatzis (2013, p.141) points out, “without surviving, there can be no thriving.”

As with the PEA, while negative emotion is a necessary component of the NEA, alone it is not sufficient to constitute the NEA state. In conjunction with negative emotion, the NEA is also characterized by arousal of the sympathetic nervous system (SNS). The SNS is associated with the human stress response and supports defensive strategies in response to experience of negative emotions. The immobilization functions of the SNS have been found to suppress our ability to engage in effective communication due to limiting facial expression, eye gaze, hand gesture, and listening abilities (Porges, 2003). In contrast to the positive health benefits associated with positive emotions and PNS arousal, prolonged periods of negative emotion and SNS arousal can be harmful to our health and wellbeing (McEwen, 1998).

The SNS is aroused when we feel that we are in physical danger, when we feel something is important, something is uncertain, or we are being evaluated (Segerstrom and Miller, 2004). Importantly, these events do not actually need to occur to arouse the SNS; humans can arouse the SNS merely by anticipating one of these conditions, e.g., anticipating the possibility of being evaluated by someone else (Sapolsky, 2004; Segerstrom and Miller, 2004). With this in mind, the process of creating a vision based with an ought self (security needs, strong ought’s, and loss/non-loss situations) almost certainly arouses the SNS.

The final layer of the NEA is the neurological activation of areas associated with the TPN. The TPN is primarily comprised of parts of the dorsal attention system (Fox et al., 2005), the frontoparietal control network (Vincent et al., 2008), and the ventral attention network (Fox et al., 2006; Kubit and Jack, 2013). The TPN is activated by tasks requiring focused attention, working memory, logical reasoning, mathematical reasoning, and causal/mechanical reasoning (Shulman et al., 1997; Duncan and Owen, 2000; Fox et al., 2005; Owen et al., 2005; Van Overwalle, 2011). Using the TPN enables us to make decisions, solve problems and focus – functions that appear critical in threat situations associated with the SNS and NEA.

The relationship between the SNS and the TPN appears to be less clear cut than that between the PNS and the DMN. While there appear to be few instances (if any) when a person would be in the SNS and the DMN, we do believe it is possible to experience positive emotions and PNS arousal associated with tasks that require the TPN, e.g., data analysis, solving equations, etc. While the relationship between the NEA and TPN has not yet been systematically tested, there is a growing body of evidence that these two constructs are tightly coupled (Matthews et al., 2004). For example, negative emotions have been found to enhance memory accuracy (Kensinger, 2007) – a task associated with the TPN. Negative emotions have been linked to paying greater attention to detail and focusing on the task at hand (Luce et al., 1997) – also functions of the TPN.

In sum, the NEA is a psycho-physiological strange attractor that is derived from unique combinations of negative affect, SNS arousal, and activation of parts of the TPN. While the NEA offers some benefits, it elicits a prevention focus and narrows our range of attention. Given this, we propose the following:

Proposition 2: The NEA is detrimental to developing a vision based on the ideal self.

### PEA–NEA in Three Dimensions

**FIGURE 1:**
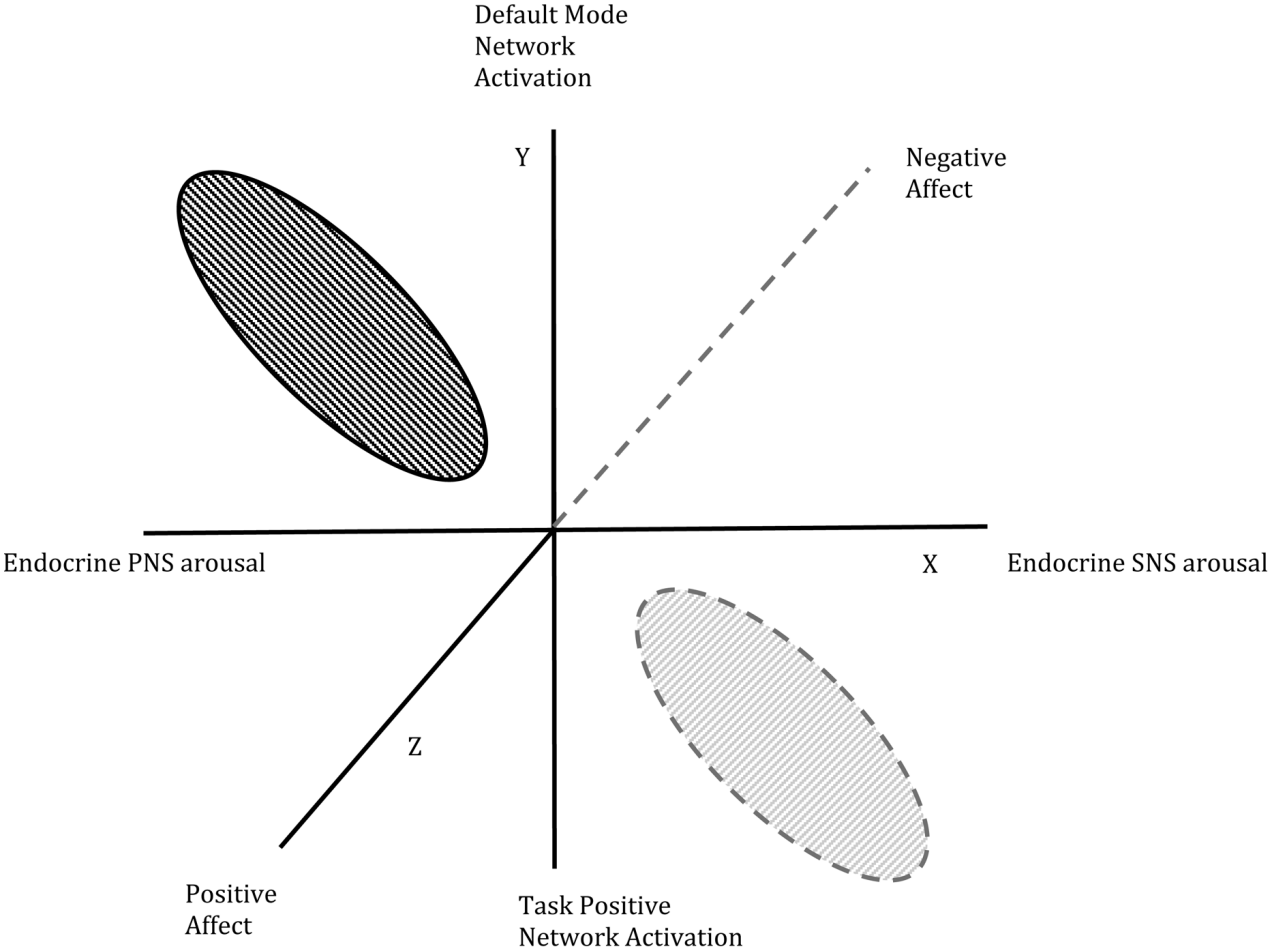
**Graphical representation of the positive emotional attractors (PEAs) and negative emotional attractors (NEAs) in intentional change theory**.

The relationship between the three dimensions that characterize the PEA and NEA states is visually depicted in **Figure 1**. This figure extends the work published by Boyatzis (2013) by re-conceptualizing the three dimensions to include neurological activation. In this reworked model, the Z-axis represents the intensity of negative to positive emotion; the Y-axis represents the activation of the TPN versus the DMN; and the X-axis represents the arousal of the PNS versus the SNS. One point of clarification is necessary regarding the depiction of positive to negative affective. While some scholars claim that positive and negative affect are two separate dimensions (Cacioppo and Berntson, 1994); other contend that positive and negative emotions can be treated as polar opposites. For example, the Circumplex Model of Emotions (Posner et al., 2005) claims emotions consist of arousal and valence. Arousal represents the vertical axis and valence represents the horizontal axis, with the center of the circumplex representing neutral valence and medium levels of arousal. Similarly, the evaluative space mode of emotions (ESMs), a counter proposal to the circumplex model, also contends “that positivity and negativity have antagonistic effects. Positivity fosters approach; negativity fosters avoidance…. Though positivity and negativity may often be characterized by reciprocal activation, they may also be characterized by uncoupled activation, coactivation, or coinhibition.” (Larsen et al., 2001, p. 686). The same authors went on to summarize, “most of our data are consistent with the circumplex prediction that polar opposite emotions are mutually exclusive.” (Larsen et al., 2001, p. 693).

In critiquing the affective literature, Russell and Carroll (1999) argued that an orthogonal dimension of degree of “activation” was needed in affective models. This claim was further supported by Posner et al., (2005) with the development of the Circumplex Model of Emotions and was also the same position taken by Gottman et al. (2002) in the creation of a mathematical model of strange attractors describing the emotional states of married couples. In the model depicted in **Figure 1**, the intensity or activation of affective arousal appears as an expression within the positive versus negative emotional arousal, endocrine, and neurological axes with low levels of arousal closer to the origin and high levels of arousal far from the origin.

These three dimensions differ from the other two models using strange attractors to depict emotional states (i.e., the Fredrickson and Gottman models). Using a concept from complexity theory, strange attractors were defined by Ed Lorenz in 1963 as something that pulls other things, in our case people’s behavior, attitudes, and feelings toward and around them, pulling them into the center. In contrast, a limit point cycle attractor pulls all in its presence into a vortex and a center (Casti, 1994). In this PEA/NEA model, once caught in the pull of an attractor, a person’s mood, state, feelings, thoughts, and behavior cycle within a self-perpetuating loop. It takes a tipping point to move the state into the pull of the other attractor. The axes of the model explain how an experience would have to change to cause a phase transition (also from complexity theory) and therefore create a tipping point in the person’s or the social group’s state.

The Fredrickson and Losada (2005) model uses team advocacy versus inquiry and self versus other as the two other axes. Meanwhile, the Gottman et al. (2002) model uses positive and negative affect and intensity of affective expression as two dimensions, but had congruence of influence styles of the husband and wife as the third dimension. Beyond their formulae, Gottman et al. (2002) did report that prediction of marital processes and outcomes was based on a balance of three “spaces” which included a physiological response of each of the members of the couple.

The tipping point between these two states, the PEA and NEA, occurs when affect is balanced between positive and negative and SNS to PNS arousal and activation of TPN to DMN are close to neutral. Intensity on all three axes must be lowered because at high intensity conditions or higher salience, perceptions will be flooded and it becomes difficult for an alternative to be seen, experienced, or even considered.

These differences in our model are particularly important in leadership and organizational settings. The physiological axis we propose helps to predict what conditions will enable or allow a person to be adaptive and open to others. Whether this involves customers, clients, patients, or students, being open to hearing their concerns and desires is essential for an effective sales or helping process. For those in management or leadership positions, this dimension helps us to understand why focusing on problems or tasks can seem to concentrate people’s attention, but may be doing it in a manner that arouses an NEA state and, therefore, closes a person emotionally, perceptually, and cognitively to alternatives. Such a result is often the opposite effect desired by the leader.

Further, since both NEA and PEA states are needed (the former for surviving and the latter for thriving), a model which helps a leader understand how to create conditions for a possible tipping point, and/or invoke one, is vital to handling complex challenges in competitive markets. During times of crisis or conflict with threatening potential consequences, awareness of the PEA and NEA states, the tipping points, and how to navigate among them can guide a leader to addressing challenges but doing so in a manner that is motivating and engaging for those around him or her.

### Role of the Positive Emotional Attractor in Visioning

In summarizing the discussion above, we believe that in order for a person, team, or organization to discover or articulate a vision based on the ideal self, they must be in the PEA. Discovering an ideal self requires efficacy, hope, and openness (Boyatzis and Akrivou, 2006). It requires people to dream, imagine future selves, and to be excited about these images. When in a NEA state, we cannot access these emotions firstly because the NEA is characterized by negative emotions and the SNS and, secondly, because the NEA includes activation of the TPN, which narrows our focus and limits our ability to think beyond our current situation.

Due to the self-reinforcing nature of strange attractors, we believe that as a person, team, or organization moves closer toward articulating an ideal self vision, the intensity of the PEA (positive emotion, PNS arousal, and DMN activation) increases (see also Fredrickson, 2001, 2004). While we might occasionally switch to the NEA during the early stages of articulating an ideal self based vision, in order to arrive at an ideal self that resonates with the person, team, or organization, the PEA must be the dominant state.

It is possible that a person perceives or believes that he or she has a vision for a desired state in the future that emerges from the NEA. We contend that such a supposed vision is emanating from an ought self, not an ideal self, and carries with it emotional obligations that are stressful to the person, invokes more SNS (and therefore triggers the NEA,) and further decreases openness to new ideas or the emerging of alternative elements of a desired state. The person’s vision, in this situation, is limited and could even be said to be constrained with a prevention focus and desire to avoid aspects of a state. On the other hand, being in the PEA state allows a person to be more open to new ideas and scan the environment for different, unexpected cues and information. This means that being in the PEA can allow a person to consider a vision, and in coordination with others a shared vision.

The literature on goal orientation suggests that a focus on specific goals may arouse the NEA and block openness to new ideas. A performance goal orientation with an emphasis on specific targets has been shown to invoke avoidance goal orientation and lower performance (VandeWalle et al., 1999). In contrast, a learning goal orientation, which is about novelty, experimentation, and learning, has been shown to enhance performance (VandeWalle et al., 1999). This could be a result of arousing the NEA with a performance goal orientation versus the PEA with a learning goal orientation. The possibility of context being a factor resulted in a comprehensive study showing that a performance goal orientation and specific goals enhanced performance when the tasks were routine but not when learning or adaptation was needed (Seijts et al., 2004). Tracking students in a statistics course over a semester revealed that time pressure aroused negative emotions and reduced the drive to mastery and eventual performance (Beck and Schmidt, 2013). Howard (2015, in this special topic) showed that the portion of a coaching session with mid-career dentists (average age 49) devoted to planning what the person would do differentially in the coming year and setting goals resulted in a dramatic reduction in positive affect and an increase in negative affect in the coaching conversations, regardless of whether the overall coaching condition was more PEA or NEA oriented. Similarly, Fisher et al. (2013) showed that people with dispositional performance goal orientation responded to an increase in task importance with greater negative and weaker positive emotions.

Proposition 3: In order to create a vision based on an ideal self a person must be in the PEA.

### Balancing the PEA with the NEA

While we believe that effective visions are created and pursued when primarily in the PEA, the NEA also plays an important role, particularly in moving a person from vision to action in the later stages of the visioning process. The NEA plays three key roles in visioning: (1) it activates the organism; (2) it provides a balance for the negative effects of excessive optimism; and (3) it encourages people to stretch and/or develop themselves (Norem, 2001). The key variable of interest here is the balance between the PEA and NEA. As discussed earlier, we know that negative emotions are stronger than positive emotions (see Baumeister et al., 2001 for a thorough review); thus, the impact of NEA experiences are stronger than PEA experiences. What we are less certain about is how much stronger negative emotions are than positive emotions. A number of positivity ratios can be found in the literature including Gottman’s (1994) 5:1; Fredrickson and Losada’s (2005) 3:1, however, a recent critique of Fredrickson and Losada’s ratio (Brown et al., 2013) has raised a fresh debate as to the relative strength of these two affective states (see also Fredrickson, 2013). However, regardless of the exact ratio, we know that for benefits of the PEA to manifest, a person, team, or organization must spend significantly more time in the PEA than the NEA. Conversely, the benefits (and costs) of the NEA can be realized in a relatively short time span (e.g., Cameron, 2008).

Higgins early work on regulatory focus suggests that it is possible for people to experience negative emotions but maintain their promotion focus. Specifically, dejection-related negative emotions such as disappointment, dissatisfaction and sadness can be experienced as a result of the absence of positive outcomes even when a person has a promotion focus. Thus, it follows that NEA experiences characterized by dejection-related emotions can be beneficial to the creation and realization of an ideal-self vision, however, only when appropriately balanced with the PEA. In contrast to dejection-related emotions, agitation-related emotions such as fear, threat, and restlessness move a person from a promotion focus to a prevention focus. We believe that these types of NEA experiences are not only not beneficial to creating and pursuing an ideal self vision, but also actively prevent a person, team, or organization from doing so.

In sum, developing an ideal-self based vision requires a person, team, or organization to be in the PEA. The NEA also plays an important role in enacting and pursing a vision. However, due to the relative strength of negative emotions over positive emotions, in order to successfully develop and pursue an ideal self based vision, a person team, or organization must spend significantly more time in the PEA than the NEA. Additionally, time spent in the NEA should be characterized by negative emotions that allow the individual to remain in a promotion-focused regulatory state.

Proposition 4: Both PEA and NEA are required in order for an ideal self based vision to lead to sustained desired change; however, a person must spend significantly more time in the PEA than in the NEA.

## Discussion

In the previous sections, we developed the rationale as to how a personal vision is based on a person’s ideal self and is necessary to lead to sustained, desired change. The requirement of a person being in the PEA to contemplate and frame a personal vision was explained, as well as how discussing one’s aspirations, hopes, and a vision can tip a person into the PEA state. We also explained why the NEA state is required for action, but to sustain any effort at change, a person must likely venture into the PEA more frequently than the NEA and spend more time in the PEA state. At the individual level, the ideal self is often compromised and suppressed by a person’s ought self or multiple ought selves.

Further, we explained how the PEA and NEA states are a result of three dimensions: positive versus negative affect, physiological arousal in terms of hormonal arousal and activation of specific neural networks. Through the dynamics of emotional contagion, we now describe how one person’s vision can become a shared vision among two or more people.

### Emotional Contagion and Developing Shared Vision

There appear to be multiple mechanisms by which one person’s dreams, emotions and PEA/NEA mood state could jump to another person, and quite literally infect them. Beginning with the neuroscience perspective, mirror neuron networks allow us to mimic the actions of others (Iacoboni, 2009), leading to a convergence of emotional states. The causal path implies that once we act in a certain way, we tend to feel the emotions of the original actor. Social or behavioral contagion may be slower but has an important effect on others nonetheless, as shown by Fowler and Christakis (2008, 2010) in epidemiologically studying the spread of new or changed behavior among social networks. Most psychologists would conclude that such contagion is caused by verbal and non-verbal imitation processes driven by social comparison processes or role modeling effects (Elfenbein, 2014).

Neuroscience would suggest that direct brain-to-brain communications is not only possible but likely and faster than the path through mirror neuron networks (Lewis et al., 2000; Decety and Batson, 2007). As Decety and Michalska (2010) showed, the brain has at least two different neural circuits that can involve empathy (i.e., perceiving the feelings or emotions of another). In this research, one version of empathy is embedded in the prefrontal cortex and overlaps with a number of regions of the brain in the TPN. The other version of empathy appears embedded in parts of the DMN, which they refer to as a hemodynamic, sympathetic network. The former allows empathy through self-reference, and the latter allows empathy that seems focused on the other person.

As a result of a series of neurological studies of charismatic leaders with vision, Waldman et al. (2014) have articulated a causal path that creates the “shared” vision. They claim that emotional equanimity and empathy lead to a balancing of positive and negative visionary communication, which in turn causes reflective and mirrored contagion among a pair or group of people (Waldman et al., 2014). Hazy and Boyatzis (2015) presented a mathematical model predicting that emotional contagion of PEA states, both neurologically and through mirroring and mimicry, would lead to creation of proto-organizing forces of people with similar valences. Regardless of the specific mechanism, the contagion appears to occur and be a force for change and adaptation in relationships or a force that dampens inhibition and retreat from desired change.

### Relationships Matter

Because of the dynamics of emotional contagion, the quality of relationships matter in determining effective leadership, engagement, and organizational citizenship. While the debate continues as to whether transformational leadership is sufficient for effective organizational performance, it appears that the quality of perceived relationship between the leader and followers mediates follower performance and citizenship (Wang et al., 2005).

In the papers in this special topic, properties of relationships that appear to be important in this causal sequence are the degree of shared vision, shared compassion and shared positive mood. Of those, shared vision consistently is the strongest indicator of a high quality relationship. The observation from these studies speaks to the transformative nature of special relationships. The shared vision in these relationships, we believe, engaged, or amplified the PEA state and the resulting openness to new ideas, people, and moral concerns.

### Too Much Vision and PEA

The effects of too much NEA are evident in experienced stress, health disorders, and public health problems (e.g., obesity, sleep deprivation, etc.). The result is a relative lack of openness to new ideas in organizations and a lack of innovation and adaptability. Too much NEA brings leaders into dissonance and disrupts relationships. It also results in the few number of ineffective leaders (Goleman et al., 2002) and decreased engagement of people in their work organizations. Even observing someone else’s anger, which will cause emotional contagion of NEA, reduces a person’s ability to be creative in problem solving (Miron-Spektor et al., 2011). The antidote is to encourage people to spend more time in the PEA. But we contend that it may be more important to help people experience multiple moments of PEA each day rather than attempting to spend prolonged periods of time in the PEA.

Research highlights the dangers of too much PEA (Boyatzis, 2013). Competition neglect, not paying attention to competitor’s innovations or progress, can be a serious consequence to spending too much time in the PEA (Camerer and Lovallo, 1999). If a strong shared vision becomes coupled with a shared belief in elitism or exceptionalism, it may lead to an overconfidence bias (Camerer and Lovallo, 1999). In an analogous manner, people high in optimism appear to make poor investment decisions by ignoring bad news and not selling stocks at a better time (Gibson and Sanbonmatsu, 2004).

### Contributions and Findings from Papers in this Special Topic

The papers in this special topic address many of the ideas presented in this paper. In health care, Quinn (2015, this issue) shows that physician leadership, as measured through organizational citizenship behavior, was predicted by emotional and social competencies, but it was mediated by the degree of PEA in terms of perceived shared vision and compassion in their relationships to others in the hospital. Meanwhile, Howard (2015, this issue) reveals that coaching mid-career dentists to the PEA engages significantly more positive affect than coaching to the NEA. Dyck (unpublished doctoral dissertation) reported that PEA behavior as coded from videos of interaction of medical students with standardized patients predicted the standardized patient’s scores of the medical student’s performance, which, by the way, was negatively affected by MCAT scores. Khawaja (2010) tested a variety of factors thought to be related to doctor-patient relationships in the medical literature. He reported that treatment adherence for Type II diabetics was predicted by many of these variables, but they were fully or partially mediated by the patient’s perception of the degree of shared vision with the doctor.

In family businesses, shared vision makes a difference in many aspects of leadership and performance. Overbeke et al. (2015, this issue) reports that daughter succession in family businesses, even in the presence of sexist family beliefs, is predicted by two factors: the daughter’s efficacy and the existence of a shared vision between the daughter and her father. Neff (2015, this issue) shows how shared vision is the strongest of five factors predicting financial performance of family businesses and their relative performance compared to competitors over 5 years. Miller (2014, this issue) expands on these two studies and shows that leadership development of the next generation in family businesses and shows that shared vision is a major factor in family business climate, which predicts leadership development.

In management, Thornton (2015, this issue) shows that shared vision as a component of perceived PEA mediated all individual variables, including conscientiousness and efficacy in predicting each of four types of corporate social responsibility: economic, discretionary, legal, and ethical. Clayton (2015, this issue) shows that successful mergers and acquisitions, as predicted by degree of championing behavior, is driven by two factors: autonomous motivation and perceived shared vision. Perceived shared vision was the strongest predictor of autonomous motivation as well. Additionally, Babu (2015, this issue) compared superior performing community college presidents with average performers and found passion and vision to be differentiators.

In organizations that others see as having a strong vision and higher purpose, Berg (2015, this issue) reports that high performing executives appear to think about their work and vision (i.e., purpose) in two distinct ways. Some see it in terms of goals and instrumental activities that will speed or enhance goal attainment. Others see a bigger picture, one that seems to transcend even the company, to a greater good for society. Meanwhile, Babu (2015, this issue) showed how more effective community college Presidents talked a lot about the vision and larger purpose than less effective community college Presidents. Hartz (unpublished doctoral dissertation) shows that the manager or leader’s degree of communicating a shared vision effects the engagement of their subordinates in manufacturing companies. Shared vision was a major factor in university investment committees’ commitment to learning and effectiveness of their knowledge management (Lord, 2015, this issue).

In the technical occupation realm, Buse and Bilimoria (2014, this issue) show that vision, hope, and a sense of purpose are key drivers in women being engaged and committed to technical careers. Meanwhile, Pittenger (2015, this issue) shows that emotional and social intelligence competencies predict organizational citizenship of IT managers, but it is fully mediated by the degree of shared vision and other elements of the PEA perceived in their relationships. Mahon et al. (2014, this issue) show, in technical knowledge worker teams, shared vision is an important antecedent of organizational engagement, enhanced by the emotional intelligence (as rated by others) of the technical works.

In coaching with the PEA focusing on personal vision, Passarelli (2015, this issue) shows that it is effective, even 30 min of it, in activating regions of the brain in the DMN, as contrasted to 30 min of NEA coaching focusing on obligations and commitments. She also discusses mental contrasting and why vision can sometimes not be sufficient for sustained action toward that vision. Finally, although not in this issue, we also learned that the quality of a relationship (i.e., perceived shared vision, compassion and positive mood – the PEA) between bank executives and an executive coach enhances the association of emotional and social intelligence on bank executives’ leader effectiveness, in terms of performance and engagement (Van Oosten, unpublished Ph.D. dissertation).

### Implications and Future Research

This paper offers three key practical implications. First, if a person, a team, or an organization are going to invest in creating a vision, they should make sure it is based on an ideal self rather than an ought self. This would require dialog among a wide spectrum of stakeholders and people within the organization, especially among those representing diversity in all differences. This means, the person, team, or organization must have a clear and shared understanding of what they value.

Second, we recommend getting oneself, a team, or organization in the PEA before working on the vision. Arousing the appropriate neural and hormonal states is important so that emotional contagion can help spread the PEA state and also to build a stock of PEA in order to buffer the NEA that may occur later in the visioning process as a person moves from vision to action. Examples of how to arouse the PEA include discussing the purpose of the organization, shared dreams or prospection of what one might become in the future, as well as discussing PEA components, like core values. Additionally, at the individual level, gratitude exercises are a powerful and fast way to evoke positive emotion and arouse the PEA.

Third, the axes of the PEA and NEA model may not be orthogonal. They may be oblique which could be clarified by research in the coexistence of the dimensions. At the same time, research is needed to determine the nature of when (or in terms of the three dimensions, where) tipping points may occur between the two attractors or states.

Finally, we emphasize the need to be cognizant of the balance between the PEA and NEA. Dreaming and visioning are of little long-term benefit to a person, team, or organization if the process does not eventually lead to action. While the PEA should dominate the early stages of vision development, the NEA will be required in the later stages. Leaders must be aware of the stronger effects of the NEA. Arousal of the NEA should be both less frequent and less intense that PEA arousal to maintain an effective balance between these two states. The collection of articles in this *Special Issue* will invoke many ideas for future research studies. These willbe explored in the various papers, however, here are a number of studies we believe need to be done to continue this line of inquiry. The impact of having a personal vision on an individual, psychologically, physiologically, behaviorally, and in terms of their key relationships should be studied. The same is true for shared vision on the people in the dyads, teams, organizations, communities, or countries. The specific processes that lead to creation and sustaining of a “shared” vision should be studied.

At some point, it would be useful to establish whether being in a PEA state enables a person to articulate a vision (or collectively a shared vision), or having a vision/shared vision enables the PEA state, or both. If both causalities occur, then the differential antecedents and consequences should be examined. Although not related to PEA and NEA, specific research needs to help establish the relationships between the neural TPN and SNS, as well as neural DMN and PNS. Emotional contagion is a key process in experiencing and sustaining a shared vision. The specific causal processes should be examined.

Beyond LMX studies of leadership effectiveness, engagement and citizenship should include quality of one’s (or the collective’s) relationships, or relational climate as a mediator or moderator. Doing so will reveal processes not considered prior to these studies. Such research would help invoke questions about whether there are other characteristics of effective relationships beyond shared vision, compassion, and positive mood. Given the eruption of controversy about the Fredrickson and Losada (2005) positivity ratio but the validity of the Gottman et al. (2002) and other selected studies, the dosage of PEA should be examined. We need to understand what a desirable ratio would yield the appropriate balance for people and collectives, and how that ratio might vary in various situations and relationships. Of course, a theme throughout all of this work is a focus on the PEA. That said, we need to better understand the role of NEA in our survival and how and when being defensive may be helpful.

## Conflict of Interest Statement

The authors declare that the research was conducted in the absence of any commercial or financial relationships that could be construed as a potential conflict of interest.
